# Intra- and Extra-Articular Double-Level Osteotomy for Pediatric Malunited Proximal Phalangeal Fracture: A Case Report

**DOI:** 10.7759/cureus.67229

**Published:** 2024-08-19

**Authors:** Hirotaka Akezuma, Keikichi Kawasaki, Ichiro Okano, Kazutoshi Kubo, Yoshifumi Kudo

**Affiliations:** 1 Department of Orthopedics, Showa University Northern Yokohama Hospital, Kanagawa, JPN; 2 Department of Orthopedic Surgery, Showa University School of Medicine, Tokyo, JPN; 3 Department of Orthopedics, Showa University Koto Toyosu Hospital, Tokyo, JPN

**Keywords:** extra-articular osteotomy, intra-articular osteotomy, intra- and extra-articular double-level osteotomy, phalangeal bicondylar t-shaped malunion, phalangeal bicondylar t-shaped fracture

## Abstract

A 13-year-old boy visited our clinic due to a malunion following a phalangeal bicondylar T-shaped fracture in the proximal interphalangeal (PIP) joint of his small finger. Imaging studies showed over 2 mm of fracture displacement and ulnar deviation of the radial condyle. The patient underwent surgical correction four weeks after the initial injury. The malunited fragments were reduced to their near-anatomical positions, and an extra-articular osteotomy was performed to realign the angular deformity. Solid bone union was successfully achieved eight weeks after the corrective surgery. This intra- and extra-articular double-level osteotomy is a good option for pediatric phalangeal bicondylar T-shaped malunions.

## Introduction

Treatment of malunion after complex articular fractures of the phalanx is challenging, since the malunion is often associated with avascular osteonecrosis and/or proximal interphalangeal (PIP) joint contracture [1]. For extra-articular malunion, extra-articular osteotomy is usually performed to correct the bone alignment and rotation [2]. On the other hand, intra-articular osteotomy is utilized for intra-articular malunion with an incongruent articular surface [3].

However, complex malunion might lead to a double-level deformity, including both intra-articular joint incongruity and extra-articular malalignment. Hence, a single approach might not be sufficient to restore normal finger function in such cases. In this report, we describe a pediatric case of phalangeal malunion after a bicondylar T-shaped fracture that was successfully treated using intra- and extra-articular double-level osteotomy, and discuss the effectiveness of this technique for complex phalangeal malunion.

## Case presentation

**Figure 1:**
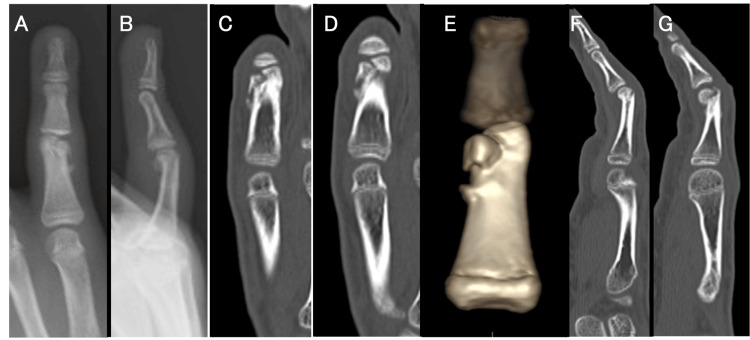
Preoperative radiographs and CT images. Radiographs (A, B) and CT scan images (C, D, E, F, G) showed that the ulnar condyle was proximally shortened, the articular step-off was 2 mm, and the radial head demonstrated 10° of ulnar deviation.

A 13-year-old boy stubbed his right little finger into a tree while riding a bicycle. He visited a nearby orthopedic clinic and was diagnosed with a proximal phalangeal fracture in the small finger. Initially, since the fracture was not displaced, he underwent conservative treatment with immobilization and splinting. The patient visited the same clinic 3 weeks later because of sustained pain in the affected finger. Although there was no rotational or sagittal angular deformity, >2 mm of fracture displacement and ulnar deviation of the radial condyle, which had not appeared on the initial radiographs, were noted on the images (Figure 1).

According to the findings on the CT scan, a displaced fracture with callus formation was observed, indicating malunion after a phalangeal bicondylar T-shaped fracture. The range of motion of his PIP joint was limited to 70° in flexion and -20° in extension, with pain. Since the articular displacement and joint malalignment were considered unacceptable, surgical correction was planned for 4 weeks after the initial injury.

**Figure 2:**
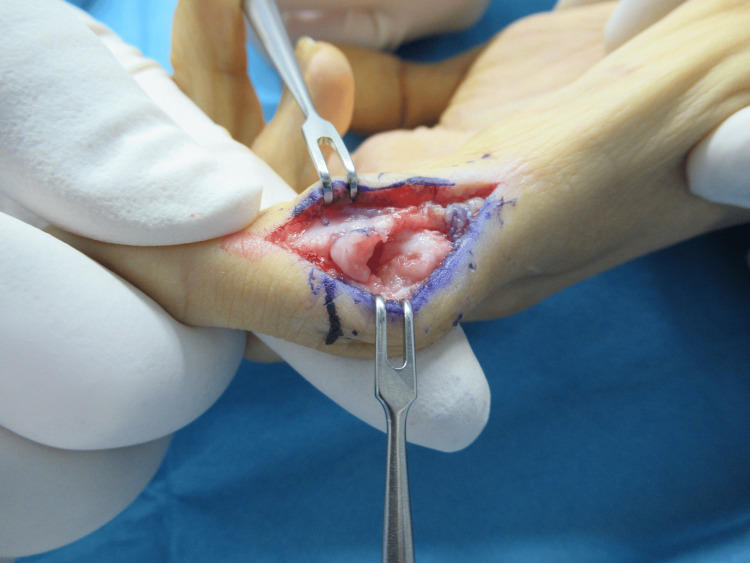
Intraoperative images. Intraoperative images showing the shortened ulnar condyle stretched distally to improve the congruity of the joint's articular surface. Next, an ulnar-side open-wedge extra-articular osteotomy was performed to correct the ulnar deviation.

During the operation, the proximal phalanx was exposed through a mid-lateral incision on the ulnar side. First, intra-articular osteotomy was performed using a chisel along the fracture line under direct visualization of the joint and with fluoroscopic guidance. The malunited fragment was reduced to the anatomical position and temporarily fixed with Kirschner wires (K-wires) under fluoroscopic guidance. Then, an ulnar-side open-wedge extra-articular osteotomy was performed using a chisel with fluoroscopy (Figure 2).

**Figure 3:**
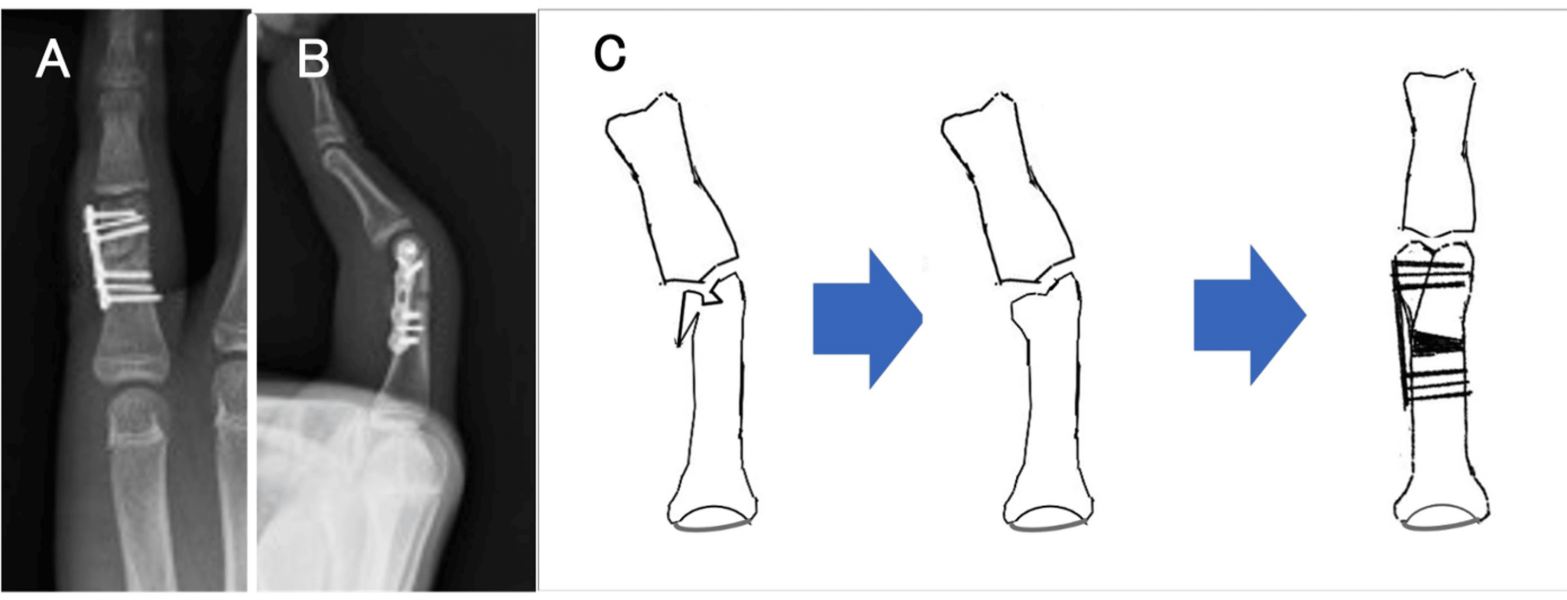
Intraoperative images. A small fragment locking plate was applied to the ulnar side of the phalanx and fixed with screws and additional Kirschner wires. (C) Graphical illustration of this procedure. Image credits: Hirotaka Akezuma.

A small fragment locking plate was applied to the ulnar side of the phalanx and fixed with screws and additional K-wires. Cancellous bone from the distal radius was grafted at the open-wedge osteotomy site (Figure 3).

**Figure 4:**
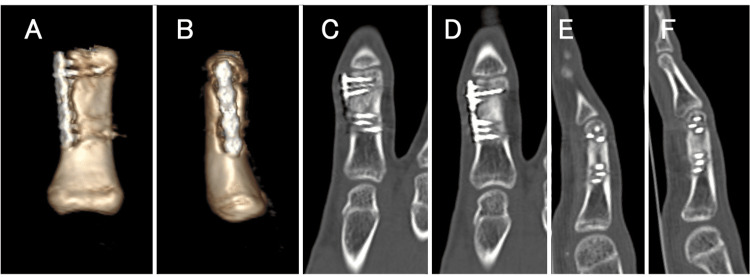
CT images at the three-month follow-up. (A, B, C, D, E, F) CT images at the three-month follow-up showed the ideal position of the proximal phalanx.

Functional rehabilitation was initiated immediately after the surgery. Buddy taping was applied, and mild range of motion exercises were initiated on the day after the surgery. Buddy taping was continued for 8 weeks postoperatively. With this treatment, solid bone union was successfully obtained at 8 weeks after the corrective surgery (Figure 4).

**Figure 5:**
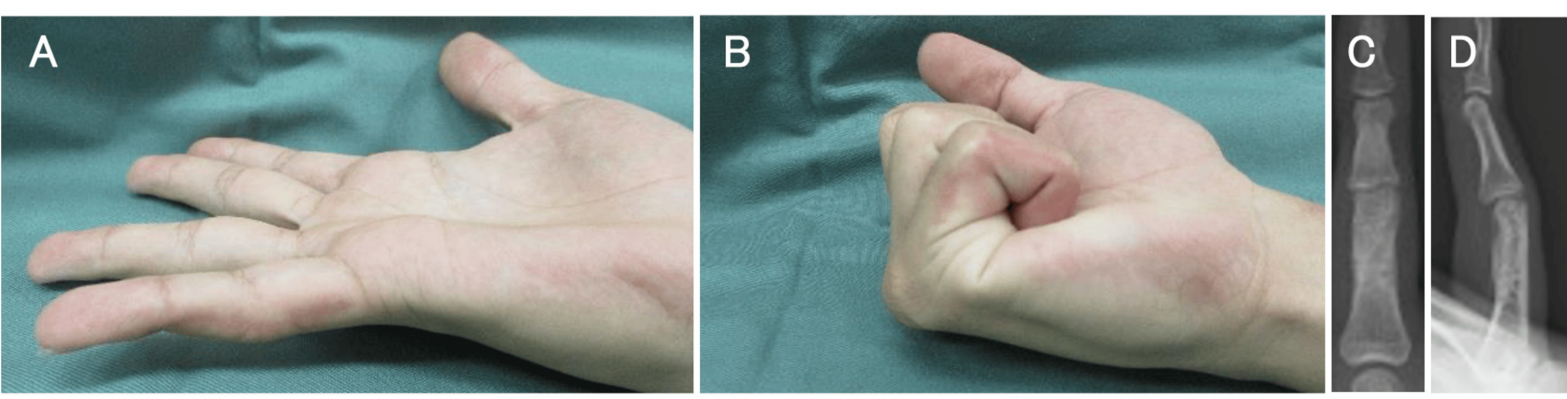
Photographs at the nine-month follow-up and radiographs at the 12-month follow-up. (A, B) Photographs showing good active range of motion of the finger at the nine-month follow-up. (C, D) Radiographs following implant removal 12 months after osteotomy, showing bone union at the proximal phalangeal fragment with good bony alignment.

The plate was removed 11 months postoperatively. At one year after the injury, the patient no longer experienced any pain and the range of motion of his PIP joint was 90° in flexion and -10° in extension (Figure 5).

## Discussion

In this report, we described a pediatric case of phalangeal malunion after a bicondylar T-shaped fracture that was successfully treated with intra- and extra-articular double-level osteotomy. Phalangeal bicondylar T-shaped fracture is a relatively rare condition, for which various fixation methods have been utilized for acute fractures. Previous reports described fixations performed using external fixators, screws, and plates [4-6]. In terms of approach, a previous report demonstrated that the fracture was exposed via a dorsal incision and reduced under direct vision between the central slip and lateral band, and then fixed with interfragmentary screws [6]. In another report, surgery was performed using a bilateral approach [5]. Unlike acute fractures, there are few specific reports of treatment for malunion after bicondylar T-shaped fracture. For simple malunions, extra-articular osteotomy for phalangeal extra-articular fracture malunion has been utilized to restore alignment of the phalanx [1, 7]. Then, the osteotomized fragments were fixed with plates and intraosseous wires. There have also been multiple reports of intra-articular osteotomy for intra-articular phalangeal hemicondylar fracture malunion [1, 3, 7-9]. Creating small bone fragments might lead to avascular necrosis (AVN) of the fragments. Also, small phalangeal bone fragments are often difficult to reduce correctly and secure during surgery [10-12]. To prevent AVN, a previous report described that condylar advancement osteotomy was useful for preventing AVN of small bone fragments [9]. However, this technique can be applied to hemicondylar fracture malunion but cannot be used for bicondylar fracture malunion.

In the present case, we used combined double-level osteotomy: intra-articular osteotomy at the fracture site and extra-articular osteotomy at the bone shaft slightly proximal to the fracture line in order to simultaneously restore both articular congruity and malalignment. In previous reports on distal radius malunion, several cases underwent intra- and extra-articular double-level corrective osteotomy and reported good results [13]. Alternatively, 3D intra-articular osteotomy was also performed for the treatment of malunion after distal radius fractures [14, 15]. However, for phalangeal malunion, a 3D intra-articular osteotomy at the fracture site appears challenging because cutting the small fracture fragments accurately is likely difficult, and the osteotomy might endanger the blood supply to the bone fragments. In contrast, during our double-level osteotomy, the operating surgeon can focus on each procedural step sequentially, intra-articular osteotomy and fixation, as well as extra-articular alignment correction, and it could be a more feasible option than 3D osteotomy.

## Conclusions

We report a case of successfully treating a pediatric phalangeal bicondylar T-shaped malunion using a combined intra- and extra-articular double-level osteotomy. This technique proved effective in addressing both intra-articular incongruity and extra-articular malalignment, thereby restoring proper finger functionality. The intra-articular osteotomy at the fracture site, combined with an extra-articular osteotomy at the bone shaft, allowed for precise realignment. Additionally, the use of a small fragment locking plate and Kirschner wires ensured stable fixation. While this approach may offer a viable solution for similar complex pediatric phalangeal malunions, further studies are needed to confirm its applicability.
